# Epinephrine penetrates through gingival sulcus unlike keratinized gingiva and evokes remote vasoconstriction in human

**DOI:** 10.1186/s12903-020-01296-z

**Published:** 2020-11-04

**Authors:** János Vág, Bernadett Gánti, Barbara Mikecs, Enikő Szabó, Bálint Molnár, Zsolt Lohinai

**Affiliations:** 1 Department of Conservative Dentistry, Semmelweis University, Szentkirályi Street 47, 1088 Budapest, Hungary; 2 Department of Periodontology, Semmelweis University, Szentkirályi Street 47, 1088 Budapest, Hungary

**Keywords:** Epinephrine, Gingiva, Blood flow, Spreading vasoconstriction

## Abstract

**Background:**

It has been demonstrated in non-oral tissues that the locally evoked vasoconstriction could elicit remote vasoconstriction. This study aimed to investigate the spreading vasoconstrictor effects of epinephrine in the gingiva.

**Methods:**

Gingival blood flow (GBF) was measured by laser speckle contrast imager in 21 healthy volunteers. In group A, two wells were fabricated from orthodontic elastic ligature and placed 2 mm apically to the free gingival margin at the mid buccal line of 12 (test side) and 21 (control side) teeth. The GBF was measured in the wells and tightly apical, coronal, distal and mesial to the wells. In group B, the wells were made on the buccal surface of the same teeth, including the gingival sulcus. Four regions were selected for measurement from the gingival margin reaching the mucogingival line (coronal, midway1, midway2 and apical). After the baseline recording, 3 µg epinephrine was applied into the test, and physiological saline into the control well. The GBF was recorded for 14 min. The gingival thickness was measured with a PIROP Ultrasonic Biometer.

**Results:**

In group A, the GBF did not increase or decrease after the application of epinephrine. In group B, the GBF significantly decreased in all regions of the test side and remained low for the observation period. The vasoconstriction appeared with delays in more apical regions (at min 1 in the coronal and the midway1, at min 2 in the midway2, at min 4 in the apical region). Similarly, the amount of the decrease at 14 min was the largest close to sulcus (− 53 ± 2.9%), followed by the midway1 (− 51 ± 2.8%) and midway2 (− 42 ± 4.2%) and was the lowest in the apical region (− 32 ± 5.8%). No correlation was found between GBF and gingival thickness.

**Conclusion:**

Epinephrine could evoke intense vasoconstriction propagating to the mucogingival junction, indicating the presence of spreading vasoconstriction in the human gingiva. The attached gingiva is impermeable to epinephrine, unlike the gingival sulcus.

This trial was registered in ClinicalTrials.gov titled as Evidence of Spreading Vasoconstriction in Human Gingiva with the reference number of NCT04131283 on 16 October 2019. https://clinicaltrials.gov/show/NCT04131283

## Background

Epinephrine is a widely used drug in dentistry. Its application is mainly based on its strong vasoconstrictor effect, which helps establish a bloodless operation area during surgery. If it is incorporated into the local anesthetic [1, 2], it delays the absorption of the local anesthetic into the blood [3]. Therefore, it prolongs the effect of the anesthetic solution [4, 5]. Epinephrine is also the most effective agent to keep the marginal area dry during the gingival retraction procedure [6, 7].

However, clinical observations suggest that epinephrine may cause secondary intention healing after wisdom tooth extraction [1], and systemic effect [8–10]. Increased epinephrine concentration is suspected of causing some rare neurological problems observed after local anesthesia due to the reduced local neural blood flow [11, 12]. It can also increase postoperative pain [13, 14]. Tissue injury was observed after the application of epinephrine with retraction cord in high dose (80 mg/ml) in animals [15–17]. However, the cytotoxic effect of epinephrine (5 mg/ml) was not observed on human gingival fibroblast [18]. During flap surgery, the incision severs the gingival blood supply, which takes 1–2 weeks to regenerate [19, 20]. Before revascularization would begin [21, 22], keeping the remaining vessels—including collaterals—open is essential for flap survival [23]. Epinephrine-induced ischemia may delay the opening of collaterals, which may compromise healing. Sheikh et al. [24] observed that the hypoperfusion in the skin evoked by epinephrine could rapidly spread from the site of injection to up to 12 mm away. The possible low-flow ischemia spreading into a wider area might be the mechanism behind tissue damage.

Previously, Laser Doppler Flowmetry (LDF) was applied to investigate blood flow following epinephrine administration with a local anesthetic. The submucous application of lidocaine with epinephrine (6.25−9 µg/ site) in human subjects resulted in a 46–51% reduction of gingival blood flow for more than an hour [25, 26]. Local anesthesia with epinephrine (45 µg/ site) applied before surgery resulted in a 68% reduction in blood flow of the alveolar mucosa [27], and the flap elevation did not decrease the blood flow further, indicating that epinephrine maximized the vasoconstriction. The application of approximately 2.24 µg epinephrine with retraction cord into the gingival sulcus resulted in a long term 50% reduction of blood flow of the marginal gingiva [6]. These studies are indicating a substantial and long-term local vasoconstrictor effect of epinephrine on human gingiva. However, LDF is a pointwise measurement tool; therefore, it is blind for spatial differences, and it is not suitable to reveal the remote effect, i.e., spreading vasoconstriction. Furthermore, previous studies did not test if the epinephrine could elicit vasoconstriction through the keratinized gingiva. The laser speckle contrast imager (LSCI) has been proved to be capable of capturing spatial variations in flap microcirculation without compromising temporal resolution with high reliability [28–33]. Recently, the nitric oxide donor-induced spreading vasodilation was also demonstrated by LSCI in human gingiva [34].

The study aimed to investigate the local and remote effects of epinephrine on human gingival blood flow (GBF) by LSCI. A secondary aim of the study was to explore the correlation between gingival thickness and the GBF.

## Methods

Eleven female and ten male volunteers were involved in the study with a mean age of 28.3 ± 6.17 between 23–43 years. The inclusion criteria were good general health, no smoking, no regular medication and at least 4 mm of keratinized gingiva at the upper front teeth. The exclusion criteria were pregnancy, breastfeeding, gingivitis, caries and filling or prosthetics rehabilitation at the upper front teeth. The ethical guideline was followed according to the Declaration of Helsinki and the approval was granted by the National Public Health and Medical Officer Service (20104/2017/EÜIG). The ClinicalTrials.gov identifier reference number for the study is NCT04131283. Our study adheres to CONSORT guidelines. The investigation was done in the Department of Conservative Dentistry at Semmelweis University. The enrolment was done between the 10th of October 2019 and the 20th of October 2019. The study was finished on 1st January 2020.

The subjects were prohibited from eating, drinking or brushing their teeth 60 min before the measurement to avoid any stimuli affecting the GBF. For 15 min before the measurement, the subjects were resting in a dental chair in a supinated position. The room temperature was controlled and kept between 24 and 26 °C. Blood pressure (Omron M2 Intellisense, Omron Healthcare Inc., Kyoto, Japan) and gingiva temperature (infrared clinical thermometer Ri-thermo® N, Rudolf Riester GmbH, Jungingen Deutschland) were measured at the beginning and the end of the experiment. The head and neck were fixed with a vacuum pillow to immobilize the patient’s head during the measurement. The lips were retracted with OptraGate (Ivoclar Vivadent AG, Lichtenstein) and the jaw was stabilized using a silicone bite.

Each participant was randomly assigned into either group A or group B by using a computer algorithm according to the simple randomization procedure. The volunteers had no information about in which group they were allocated. The experimental preparation and the measurements were made by the same investigator for all subjects. Epinephrine (Tonogen 1 mg/ml, Richter Gedeon Plc, Hungary) was applied in 3 µl (equal to a 3 µg dose) on the test side. On the control side, 3 µl physiological saline was used. Before their application, both solutions and their application syringes were preheated to 36.5 °C in a block heater (Boeco, Germany).

**Fig. 1:**
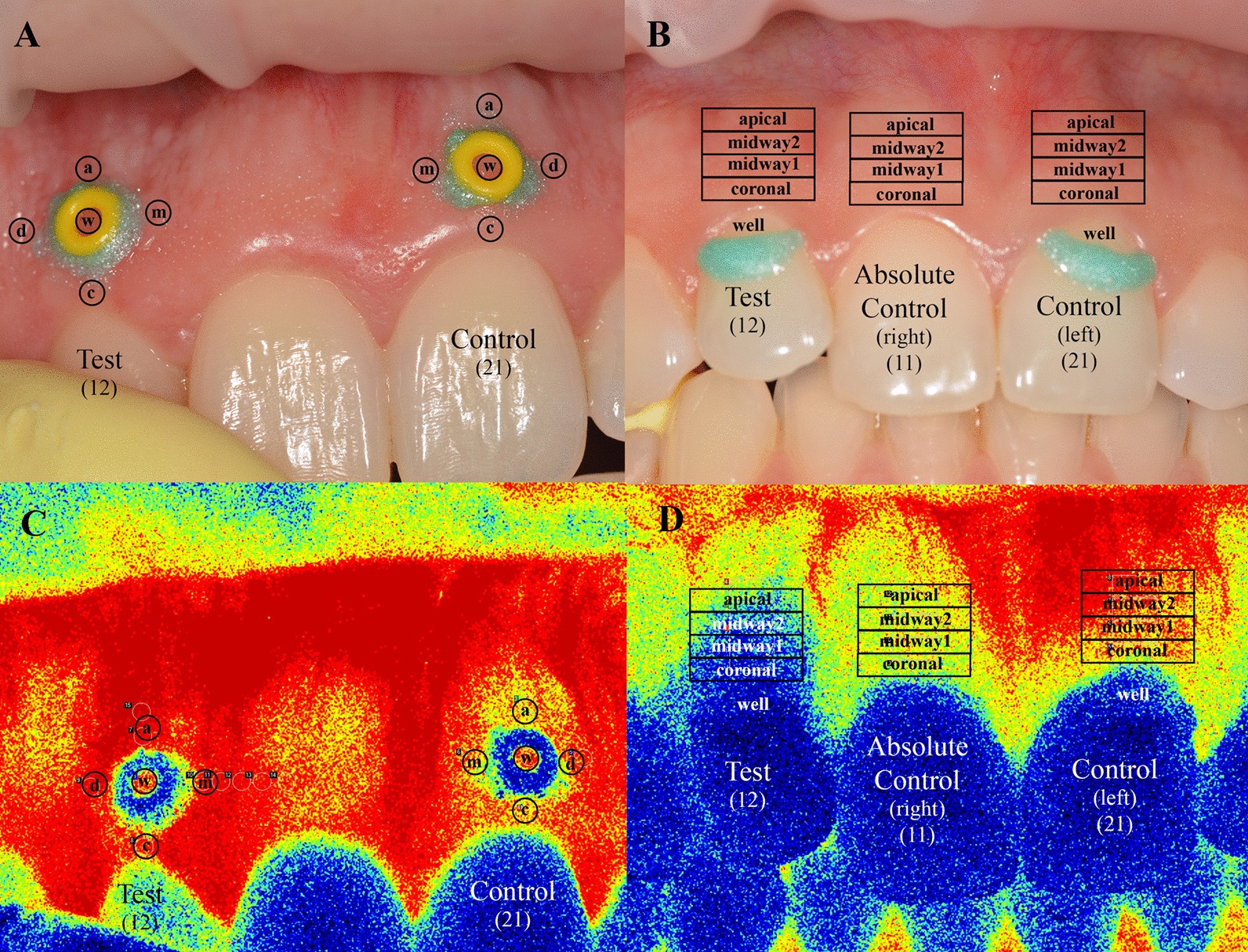
The clinical photograph shows the position of the wells and regions of interest in group A (**A**) and in group B (**B**). The laser speckle contrast images show a lack of ischemic area after the application of epinephrine in the test well in group A (**C**) and four ischemic regions above the test tooth in group B (**D**). The colors indicate the different intensity of the blood flows from the lowest to the highest in order of blue, green, yellow, red

In group A, two wells were fabricated from elastic orthodontic ligature with a diameter of 1.14 mm, and they were fixed by light-cured resin-based barrier material (Opal Dam green, Ultradent Products Inc., USA). The wells were placed 2 mm from the free marginal gingiva at the mid buccal line of the tooth on the keratinized gingiva (Fig. 1a). The test side was at the upper right second incisor (FDI #12), the control well was at the upper left first incisor (FDI #21). Ten regions of interest (ROI) were selected as follows: the well (“w”), mesial to the well (“m”), distal to the well (“d”), apical to the well (“a”), and coronal to the well (“c”) on both the test and control sides.

In group B, two wells were fabricated on the surface of the teeth. A well was made at the upper right second incisor (FDI #12) for test solution and another at the upper left first incisor (FDI #21) for saline (Fig. 1b). No well was formed at the upper right first incisor (FDI #11). The coronal border of the wells was made of light-cured resin-based barrier material on the surface of the tooth as a semicircular crest. Apically, they were demarcated by the gingival margin. The 4 ROIs were defined above each tooth investigated. Each was 1 mm height, the coronal (from the marginal gingival zenith to the first 1 mm apically), the midway1 (from 1 to 2 mm), the midway2 (from 2 to 3 mm) and the apical (from 3 to 4 mm). The regions above tooth #11 were considered as absolute control.

In both groups, after the preparation procedure, the blood flow was allowed to stabilize for 15 min. After the baseline GBF was recorded for 1 min, the epinephrine and physiological saline were applied to the corresponding wells with a Hamilton syringe (Model 75 RN SYR, Hamilton, Switzerland). GBF changes were continuously recorded for further 14 min by LSCI (PeriCam PSI HR System, Perimed AB, Stockholm, Sweden) with a 2 × 3 cm measurement area, 0.06 mm/pixel resolution and 2 images/sec. The gingival thickness was measured by PIROP ultrasonic biometer (Echo-Son, Puławy, Poland) [34, 35] at the test and control sides according to the location of the 5–5 ROIs in group A, and at the midway1 and apical regions in group B.

## Statistical analysis

The sample size was determined by experience from the previous study with very similar set-up [34]. Data are presented in the text and the figures as mean ± standard error. The changes in GBF were calculated by the difference between the laser speckle perfusion unit (LSPU) at a specific observation time point and its baseline value (dLSPU). Data were statistically analyzed by the generalized linear mixed model with time, side, region and their interactions as main factors. GBF at the test side was compared to the control side in the corresponding regions using the baseline values as a covariate. The covariate adjusts the differences in baselines between regions. During post-hoc pairwise comparison, the p-value of less than 0.05 was considered statistically significant after Bonferroni adjustment. The relationship between the gingival thickness and the GBF value was assessed by Pearson’s product-moment correlation. The analysis was carried out using IBM SPSS Statistics, Version 25 (Armonk, NY: IBM Corp., USA).

## Results

Before and after blood flow measurement, the mean arterial blood pressure did not change significantly in group A (86 ± 1.7 vs. 83 ± 1.5 mm Hg, *p* = 0.065), and significantly reduced in group B (from 82 ± 2.1 to 79 ± 2.3 mm Hg, *p* = 0.015). The pulse rate decreased significantly in group A (from 71 ± 4.3 to 65 ± 3.4 beats per minute, *p* = 0.012), but not in group B (61 ± 2.7 vs. 63 ± 2.8 beats per minute, *p* = 0.371). The temperature of the gingiva did not change significantly in group A (34.5 ± 0.43 vs. 33.8 ± 0.44 °C, *p* = 0.106) nor in group B (33.1 ± 0.42 vs. 33.1 ± 0.37 °C, *p* = 0.952) during the measurement period.

**Fig. 2:**
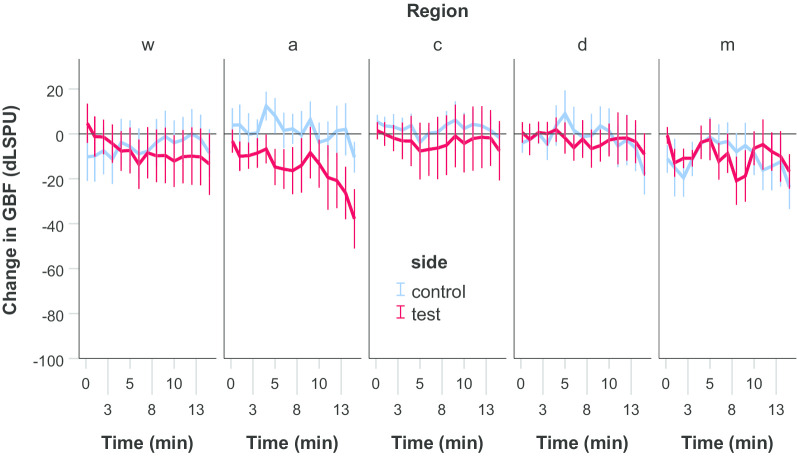
Change in gingival blood flow (GBF) from the baseline in group A. No significant differences were observed between the test and the control sides in either region

In group A (n = 11) neither the three-way interaction (side × region × time, *p* = 0.999) nor any two-way interactions (side × region, *p* = 0.352, side × time *p* = 0.244, region × time *p* = 0.956) were significantly different. Among the main effects, ‘side’ was not significant (*p* = 0.203), indicating no effect of epinephrine (test side) over the saline (control side) (Fig. 1c). On the other hand, the significant effect of the main ‘time’ effect (*p* < 0.001) indicated an overall decrease in GBF by the time on both sides (Fig. 2) regardless of regions. The mean drop was 13.0 ± 3.0 LSPU (*p* < 0.001) from the beginning to the end of the observation (min 14).

**Fig. 3:**
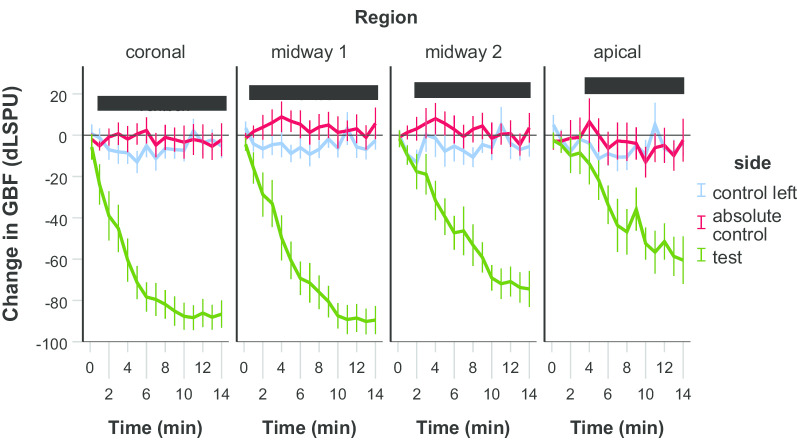
The change in gingival blood flow (GBF) from the baseline in group B. The black bars spanning the corresponding time points indicate statistical differences between the test side and any corresponding control side, *p* < 0.05

In group B, the GBF at the test side was compared to the GBF at the control side (saline application) and to the absolute control regions (no stimulus was applied) at each time-points (Fig. 3). GBF in the test side was significantly lower from min 1 at the coronal and the midway1 region, from min 2 at the midway2 region, and from min 4 at the apical region compared to control regions. Differences in magnitude and time were observed among the test regions. The changes in the GBF values of remote regions (midway1, midway2, apical) on the test side were significantly smaller compared to the coronal region. GBF was significantly smaller from min 3 to min 8 in the midway1 region (*p* values were between 0.008 and 0.045), from min 1 to min 14 in the midway2 (*p* values were between 0.001 and 0.025), and the apical regions (*p <* 0.001 at each time-points). The changes observed at min 14 was − 87 dLSPU (from 162 ± 1.9 to 75 ± 5.0 LSPU, − 53 ± 2.9%) in the coronal region, − 89 dLSPU (from 174 ± 2.0 to 84 ± 5.9 LSPU, − 51 ± 2.8%) in the midway1 region, − 74 dLSPU (from 176 ± 2.4 to 101 ± 8.7 LSPU, − 42 ± 4.2%) in the midway2 region, and − 60 dLSPU (from 186 ± 2.1 to 125 ± 9.4 LSPU, − 32 ± 5.8%) in the apical region.

The gingival thickness of the patients varied from 2.44 to 0.25 mm. No correlation was found between baseline blood flow and gingival thickness in any region or group (Pearson r ranged from − 0.42 to 0.28, *p*-value ranged from 0.13 to 0.96). In group B, where a significant ischemia was observed, no significant correlation was found between the ischemia at min 14 and gingival thickness in either the measured midway1 or the apical region (r = 0.47, *p* = 0.167, and r = 0.44, *p* = 0.208, respectively).

## Discussion

In the present study, LSCI, with the high spatial resolution, was used to capture the remote effect and the regional variation in the vasoconstriction evoked by locally applied epinephrine. When 3 µg epinephrine was applied on keratinized gingiva, neither local nor remote vasoconstriction were observed. However, the same dose applied in the gingival crevice provoked intense vasoconstriction in the tested areas. In accordance with this result, previously [6, 7], 2.24 µg epinephrine resulted in a 50% reduction in the blood flow of the marginal gingiva after application in the gingival sulcus by retraction cord. The alveolar bones are lined with different types of mucosal epithelia, having various characteristics. The attached gingival epithelium constitutes of stratified squamous keratinized epithelium, while the sulcus epithelium appears to be stratified and non-keratinized [36, 37]. The keratinized mucosa highly resembles the skin [38]. Epinephrine can penetrate through the skin only by iontophoresis [39–41]. Similarly, the keratinized gingiva may be impermeable to epinephrine, which could explain the inefficacy of epinephrine for this administration route.

Intra-arterial infusion of epinephrine decreases the blood flow in rabbit gingiva despite the blood pressure elevation [42]. The absorbed catecholamines can bind to ɑ-adrenergic receptors found within vessels, causing vasoconstriction [43–45]. This role of adrenergic receptors on the blood flow regulation was demonstrated in rat gingiva by the selective inhibition of α_1_ and α_2_ receptors [46] and by the selective α_2_ agonist in human palatal mucosa [45]. In conclusion, epinephrine could be absorbed in the gingival sulcus, and it binds to the adrenergic receptors causing vasoconstriction.

Vasoconstriction was observed in all regions apically to the sulcular application up to 4 mm. As our measurement was limited to this point, we may expect further spreading. In animal skin flap, the hypoperfusion evoked by epinephrine spread from the site of injection up to 12 mm [24].

Spreading vasoconstriction seems to depend on the innervation of the tissue. In the retractor muscle with strong sympathetic innervation, norepinephrine caused only local vasoconstriction [47]. In contrast, in the cheek pouch, which lacks sympathetic innervation [48], norepinephrine evoked propagated vasoconstriction suggesting a non-neural mechanism [49–51]. The gingiva is innervated by sympathetic nerves running with the blood vessels [52] and by parasympathetic fibers running together with sensory or motor nerves [53]. The blood flow, however, is primarily controlled by the parasympathetic innervation, and the role of the sympathetic and sensory nerve is less remarkable during physiological conditions [54–56]. It resembles more the cheek pouch model; thus, the presence of spreading vasoconstricton in gingiva might be related to the weak control of the sympathetic nerve in blood flow regulation. However, identifying the relationship between neural control and the spreading mechanism requires further studies.

The low flow-mediated vasoconstriction is a suggested mechanism that may explain spreading vasoconstriction [57]. The decreased blood flow reduces the shear stress, which results in vasoconstriction [58]. It is primarily linked to the baseline tone [59] and probably mediated by endothelin-1, endothelium-derived hyperpolarizing factor and prostaglandins [57]. The skin has a low resting blood flow**;** therefore, the effect of epinephrine on microcirculation cannot be detected by LDF and LSCI [40]. On the contrary, the gingiva seems to have a much higher resting blood flow. In this study, epinephrine caused a 54% decrease in blood flow. This is in accordance with the previous studies [25, 26]. The submucous application of lidocaine with epinephrine (6.25–9 µg/ site) in human subjects resulted in a 46–51% reduction of gingival blood flow for more than an hour. In another study [27], local anesthesia with epinephrine (45 µg/ site) was applied submucosally before surgery. It reduced the blood flow of the alveolar mucosa by 68%. After the surgery, no further drop was observed, suggesting that epinephrine already maximized vasoconstriction.

Another mechanism behind the spread of vasoconstriction could be the diffusion of epinephrine from the sulcus to the apical area. The distance between the site of application and the apical region was 4 mm in our study. The diffusion expected to have about 1.3 mm/min velocity [60], thus a remote response should have  approximately 3 min delay at a distance of 4 mm. Vasoconstriction was observed after 4 min in the apical region, contrary to the 1 min, after which it was already seen at the coronal and midway1 regions as well. The mid buccal gingival area at the adjacent incisor served as an absolute reference site without any stimulus. The coronal region here was approximately within the same distance as the apical region of the test site. Therefore, if the epinephrine could diffuse freely in all directions, a similar vascular response would be expected in the absolute control region as well. In this study, the response front spread dominantly apically (see Fig. 1D), which resembles the apico-coronal orientation of the blood supply of the gingiva [61–63]. Therefore, we assume that the spreading vasoconstriction observed was related to the blood supply orientation. Consequently, the endothelium, the smooth muscle cells of the vessels, the flow, or the nerve along the vessels may mediate this response. In order to elucidate the mechanism, further human studies are required.

In our study, no correlation was found between the baseline blood flow and the gingival thickness in either group. The LSCI detects blood movement in the superficial 0.3–0.7 mm of the tissue [64]. The superficial layers of the gingiva include the subepithelial capillary network and the small connective vessels just below that [62, 65]. The superficial, extremely dense capillary mesh is rich in red blood cells. These cells reflect most of the light for LSCI. Therefore, it is expected that blood flow from this layer contributes the most to the LSCI signal in the gingiva. According to our result, there is no difference in microcirculation between the gingival biotypes.

## Conclusions

The keratinized gingiva seems to be impermeable to epinephrine. However, its application into healthy gingival sulcus evokes powerful vasoconstriction, which can effectively spread vertically toward the mucogingival junction  opposite to supply vessels.

## Data Availability

The datasets used and/or analyzed during the current study are available from the corresponding author upon reasonable request.

## Abbreviations

GBF: Gingival blood flow
LSCI: Laser speckle contrast imager
LDF: Laser Doppler flowmetry
